# Age may be a moderator of operative time in patients undergoing transoral endoscopic thyroidectomy vestibular approach

**DOI:** 10.1097/JS9.0000000000001183

**Published:** 2024-02-12

**Authors:** Kuo-Chuan Hung, I-Ting Tsai, I-Wen Chen, Cheuk-Kwan Sun

**Affiliations:** a Department of Anesthesiology, Chi Mei Medical Center, Liouying, Tainan City; b Department of Emergency Medicine, E-Da Hospital, I-Shou University; c School of Medicine, College of Medicine, I-Shou University, Kaohsiung City, Taiwan

*Dear Editor*,

We read with interest the systematic review and meta-analysis by Oh *et al*.^1^ that compared the outcomes between patients undergoing transoral endoscopic thyroidectomy vestibular approach (TOETVA) and those receiving open thyroidectomy (OT). This study addresses a crucial topic in the field of thyroid surgery, providing valuable insights into the efficacy and safety of TOETVA compared to those of conventional OT. The meta-analysis^1^ demonstrated that TOETVA, despite having a longer operative time, may be cosmetically preferable to OT, especially for patients concerned about postoperative scarring. This advancement in minimally invasive surgery can significantly enhance a patient’s quality of life by minimizing postoperative pain and scarring. We would like to extend our congratulations to the authors^1^ on their comprehensive analysis as well as the significant contribution of their work to thyroid surgery literature.

Nevertheless, despite the authors’ report of a significantly longer overall operative time for patients undergoing TOETVA than that in those receiving OT with a weighted mean difference (WMD) of 55.19 min, the high heterogeneity (*I* ^2^=97%) of this finding remains a concern^1^. Although the authors attributed the finding to a potential variation in the underlying condition of the patient^1^, further analysis was not conducted. To address this issue, we performed a meta-regression analysis targeting the impact of age and nodule size on operative time based on the raw data of the meta-analysis^1^. The software Comprehensive Meta-Analysis (Version 4, Biostat) was used for analyzing the association as previously reported^2,3^. As illustrated in Figure 1 of our analysis, age appeared to be a significant moderator of operative time (coefficient: −2.89, *P*=0.002). Specifically, for younger patients undergoing TOETVA, the operative time was noticeably longer than that in those undergoing OT. This finding suggests that patient age should be considered when deciding between TOETVA and OT, as it may influence the duration of the surgery. Our result is consistent with that of a previous clinical study on 714 patients receiving thyroid surgery for papillary thyroid carcinoma (PTC) or suspected PTC that identified young age as a predictor of difficult thyroidectomy, probably due to tough neck tissue^4^. On the other hand, our result showed that nodule size did not have any impact on operative time in both groups (coefficient: −1.33, *P*=0.403). This finding is also supported by that of a previous study^4^. Nevertheless, it should be emphasized that other confounding factors, such as operative experience^5^, tissue laxity, and variations in histology, may also influence operative time.

**Figure 1:**
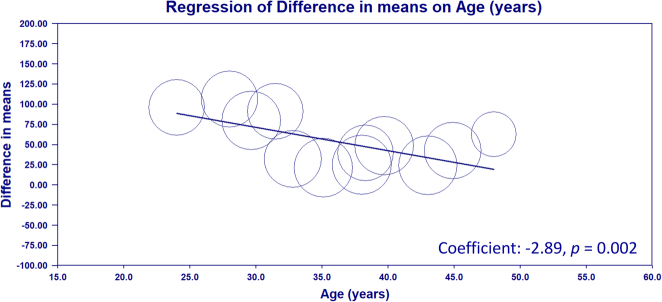
Meta-regression evaluating the impact of age on operative time between transoral endoscopic thyroidectomy vestibular approach (TOETVA) and open thyroidectomy (OT) groups. The size of the circles represents the weight of each study. The regression line indicates a significant negative association between age and operative time (coefficient: −2.89, *P*=0.002) with a younger age in the TOETVA group correlating with longer operative times compared to OT.

In conclusion, while the meta-analysis by Oh *et al*.^1^ is a valuable addition to our understanding of TOETVA; addressing these issues could shed light on the selection of patients who would benefit most from TOETVA.

## Ethical approval

Not applicable.

## Consent

Not applicable.

## Sources of funding

No external funding was received for this study.

## Author contribution

K.-C.H. and C.-K.S.: wrote the main manuscript text; I-T.T. and I-W.C.: prepared Figure 1. All authors read and approved the final version of the manuscript.

## Conflicts of interest disclosure

The authors declare no conflicts of interest.

## Research registration unique identifying number (UIN)

Not applicable.

## Guarantor

Kuo-Chuan Hung.

## Data availability statement

The datasets used and/or analyzed in the current study are available from the corresponding author upon reasonable request.

## Provenance and peer review

This paper was not invited.
